# Gene Pyramiding for Achieving Enhanced Resistance to Bacterial Blight, Blast, and Sheath Blight Diseases in Rice

**DOI:** 10.3389/fpls.2020.591457

**Published:** 2020-11-19

**Authors:** Jegadeesan Ramalingam, Chandavarapu Raveendra, Palanisamy Savitha, Venugopal Vidya, Thammannagowda Lingapatna Chaithra, Senthilvel Velprabakaran, Ramasamy Saraswathi, Ayyasamy Ramanathan, Madhavan Pillai Arumugam Pillai, Samudrakani Arumugachamy, Chockalingam Vanniarajan

**Affiliations:** ^1^Centre of Excellence for Innovations, Department of Biotechnology, Agricultural College and Research Institute, Tamil Nadu Agricultural University, Madurai, India; ^2^Centre for Plant Molecular Biology and Biotechnology, Tamil Nadu Agricultural University, Coimbatore, India; ^3^Department of Plant Breeding and Genetics, Agricultural College and Research Institute, Tamil Nadu Agricultural University, Madurai, India; ^4^Department of Rice, Centre for Plant Breeding and Genetics, Tamil Nadu Agricultural University, Coimbatore, India; ^5^Tamil Nadu Rice Research Institute, Tamil Nadu Agricultural University, Aduthurai, India; ^6^Agricultural College and Research Institute, Tamil Nadu Agricultural University, Killikulam, India; ^7^Rice Research Station, Tamil Nadu Agricultural University, Ambasamudram, India

**Keywords:** rice, multiple disease resistance, marker-assisted backcross breeding, gene pyramiding, phenotyping

## Abstract

Bacterial blight, blast, and sheath blight are the commonest diseases causing substantial yield loss in rice around the world. Stacking of broad-spectrum resistance genes/QTLs into popular cultivars is becoming a major objective of any disease resistance breeding program. The varieties ASD 16 and ADT 43 are the two popular, high yielding, and widely grown rice cultivars of South India, which are susceptible to bacterial blight (BB), blast, and sheath blight diseases. The present study was carried out to improve the cultivars (ASD 16 and ADT 43) through introgression of bacterial blight (*xa5*, *xa13*, and *Xa21*), blast (*Pi54*), and sheath blight (*qSBR7-1*, *qSBR11-1*, and *qSBR11-2*) resistance genes/QTLs by MABB (marker-assisted backcross breeding). IRBB60 (*xa5*, *xa13*, and *Xa21*) and Tetep (*Pi54*; *qSBR7-1*, *qSBR11-1*, and *qSBR11-2*) were used as donors to introgress BB, blast, and sheath blight resistance into the recurrent parents (ASD 16 and ADT 43). Homozygous (BC_3_F_3_ generation), three-gene bacterial blight pyramided (*xa5* + *xa13* + *Xa21*) lines were developed, and these lines were crossed with Tetep to combine blast (*Pi54*) and sheath blight (*qSBR7-1*, *qSBR11-1*, and *qSBR11-2*) resistance. In BC_3_F_3_ generation, the improved pyramided lines carrying a total of seven genes/QTLs (*xa5* + *xa13* + *Xa21* + *Pi54* + *qSBR7-1* + *qSBR11-1* + *qSBR11-2*) were selected through molecular and phenotypic assay, and these were evaluated for resistance against bacterial blight, blast, and sheath blight pathogens under greenhouse conditions. We have selected nine lines in ASD 16 background and 15 lines in ADT 43 background, exhibiting a high degree of resistance to BB, blast, and sheath blight diseases and also possessing phenotypes of recurrent parents. The improved pyramided lines are expected to be used as improved varieties or used as a potential donor in breeding programs. The present study successfully introgressed *Pi54*, and *qSBR* QTLs (*qSBR7-1*, *qSBR11-1*, and *qSBR11-2*) from Tetep and major effective BB-resistant genes (*xa5*, *xa13*, and *Xa21*) from IRBB60 into the commercial varieties for durable resistance to multiple diseases.

## Introduction

Rice (*Oryza sativa* L.) is considered a major staple food crop for billions of population across the globe, and it provides 23% of calories shared by different food crops (Sharma et al., 2012). Exponential growth of the world population demands an increase in rice production by 26% to fulfill calorie requirements (Khush, 2013). According to the Food and Agricultural Organization, the global rice production would have to increase by 42% over the present-day production to meet the growing population by 2050 (Ray et al., 2013). However, the yield potential is frequently threatened by various biotic stresses, mostly fungi, and bacteria. To address these problems and to increase production, developing cultivars with durable resistance is a prerequisite. The host-plant resistance can be ideally improved through pyramiding of major *R*-genes/QTLs for multiple diseases and biotic stress factors.

Bacterial blight (BB) caused by *Xanthomonas oryzae* pv. *oryzae* (*Xoo*) is a major destructive disease of rice, causing a yield loss of up to 80% depending on the severity (Kumar et al., 2012). Improving the host-plant resistance is the most efficient and eco-friendly approach, as chemical control of BB is not effective (Lee et al., 2003). The infection chain starts by entering into the plant through the hydathodes, and it reaches to xylem vessels, where the infection became systemic. Till date, 46 resistance genes have been identified from the different sources of rice (Chen et al., 2020). Of these, the *Xa4*, *xa5*, *Xa7*, *xa13*, *Xa21*, *Xa33*, and *Xa38* genes are most frequently utilized in hybridization programs for developing BB-resistant cultivars (Hsu et al., 2020). Natural allelic variations in *Xoo* challenges the resistance levels conferred by a single gene; hence, pyramiding of two or more effective resistance genes is highly essential for broad-spectrum and durable resistance to *Xoo* at field conditions. *Xa21*, a major dominant resistant gene, originated from African wild species, *Oryza longistaminata*, was observed to confer the resistance to many *Xoo* isolates (Nguyen et al., 2018). The encoding proteins of *Xa21* gene carries both leucine-rich repeats (LRR) and serine-threonine kinases, and these complexes perceives the presence of pathogen ligand on the cell surface and activates the subsequent intracellular defense response *R*-proteins (Song et al., 1995). The *Xa21* gene was physically mapped on the long arm of chromosome 11, and a highly efficient PCR-based co-dominant molecular marker (pTA 248) was developed for marker-assisted selection of *Xa21* (Ronald et al., 1992). A unique, fully recessive gene, *xa13*, was first identified in cultivar BJ1 and physically mapped on the long arm of chromosome 8 (Zhang et al., 1996). Mutations in the promoter region of dominant allele (*Xa13*) resulted in a recessive gene, *xa13*, which does not encode for a modulator for pathogen (Chu et al., 2006). Another broad-spectrum recessive resistant gene, *xa5*, was identified and mapped on the subtelomeric region of chromosome number 5 (Blair et al., 2003). Unlike other *R*-genes, the *xa5* gene encodes for a gamma transcription factor-like protein (TFIIAγ). Pyramiding of *xa5* gene with other dominant genes gives durable resistance to *Xoo* than the plants with single BB-resistant gene (Huang et al., 1997).

Rice blast, caused by *Magnaporthe oryzae* (Teleomorph: *Pyricularia oryzae*), is one of the devastating diseases of rice growing areas across the world. Yield loss is estimated to be more than 50% when it occurs in epidemic proportions (Babujee and Gnanamanickam, 2000). Similar to BB, developing of host-plant resistance is the most effective strategy for management of blast disease (Sharma et al., 2012). So far, about 100 resistance genes have been identified, and 37 of them were cloned (Zhang et al., 2019). Although several blast resistance genes have been identified, only a few of them were used in breeding programs for blast disease management in India (Singh et al., 2011). Among them, the *Pi54* gene located on chromosome 11 provides stable and durable resistance to diverse strains of *M. oryzae* collected across India (Thakur et al., 2015). The predicted proteins of the *Pi54* gene contains NBS-LRR proteins along with unique zinc finger domain (Sharma et al., 2005). During the host–pathogen interaction, the *Pi54* gene induces the synthesis of callose (β-1,3-glucan), which acts as a physical barrier by blocking the penetration of fungal hyphae (Gupta et al., 2012). A functional marker has been developed for the *Pi54* gene and used in maker-assisted selection for developing blast-resistant cultivars (Ramkumar et al., 2011).

*Rhizoctonia solani* Kühn, the causative agent of rice sheath blight disease (ShB), poses a significant impact on both yield and quality (Singh et al., 2019). Introduction of high yielding varieties and application of high doses of nitrogenous fertilizers resulted in a steep rise in incidence of sheath blight disease (Savary et al., 1997). *R. solani* Kühn is a soil-borne facultative parasite, survives as sclerotia or mycelium, or rarely as basidiospores. No varieties resistant to sheath blight were reported till date (Channamallikarjuna et al., 2010). Breeding for resistance to ShB is quite unsuccessful owing to inability to identify effective resistance sources from the available germplasm, wide-ranging host compatibility, high genetic variability, and capability of the pathogen to survive from season to season in the form of dormant sclerotia, makes additional complications in controlling the disease (Molla et al., 2020). Though qualitative resistance to ShB was not found, quantitative resistance was reported in some landraces *viz*., Tetep, Teqing, Jasmine 85, etc., (Channamallikarjuna et al., 2010; Wang et al., 2012; Yadav et al., 2015). Up to now, 50 QTLs conferring moderate resistance to rice sheath blight have been identified from the different sources of rice (Zhang et al., 2019). Among these, *qSBR7-1*, *qSBR11-1*, and *qSBR11-2* were identified in the background of Tetep (Channamallikarjuna et al., 2010) and pyramided in Pusa 6B (Singh et al., 2015). We have used these three QTLs (*qSBR7-1*, *qSBR11-1*, and *qSBR11-2*) for pyramiding in our recurrent parents to improve sheath blight resistance.

In cognizance of the above reports, the present study was formulated with the following objectives: (i) introgression of BB (*xa5*, *xa13*, and *Xa21*), blast (*Pi54*), and sheath blight (*qSBR7-*1, *qSBR11-1*, and *qSBR11-2*) resistance genes/QTLs in the backgrounds of ASD 16 and ADT 43; (ii) analysis of recurrent parent genome recovery (RPG) with a set of polymorphic SSR (simple sequence repeats) markers; (iii) evaluation of improved pyramided lines for physical resistance against BB, blast, and sheath blight diseases; and (iv) evaluation of agro-morphological and quality traits of improved pyramided lines in comparison with parents.

## Materials and Methods

### Plant Materials

The two recurrent parents, ASD 16 (ADT 31 × CO 39) and ADT 43 (IR 50 × Improved White Ponni) are popular, high yielding, and widely grown rice cultivars of South India. ASD 16 has short bold grains, while ADT 43 has medium slender fine grains. Though both cultivars are high yielding, they are highly susceptible to BB, blast, and sheath blight diseases. A bacterial blight-resistant genotype, IRBB60 harboring *xa5* + *xa13* + *Xa21* was used as donor for BB resistance genes. Tetep, a Vietnamese *indica* land race possessing blast resistance (*Pi54*) (Ramkumar et al., 2011) and moderately resistant to sheath blight, harboring qSBR QTLs (*qSBR7-1, qSBR11-1*, and *qSBR11-2*) (Channamallikarjuna et al., 2010) was also used as one of the donors for targeted transfer of blast and sheath blight resistance into the background of recurrent parents (ASD 16 and ADT 43).

### Marker-Assisted Backcross Breeding for Targeted Gene/QTL Pyramiding of Bacterial Blight, Blast, and Sheath Blight in the Backgrounds of ASD 16, and ADT 43

**FIGURE 1:**
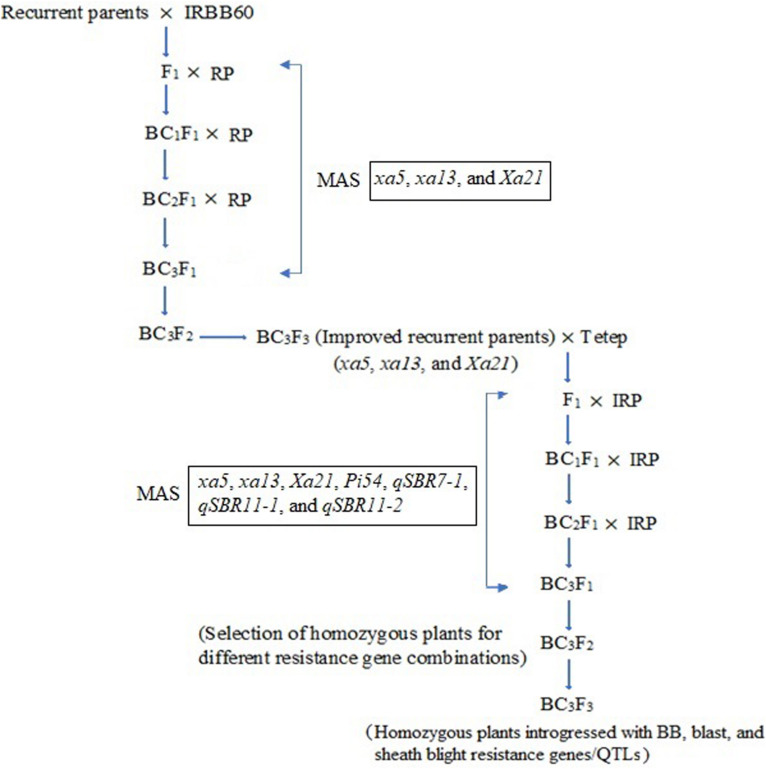
Schematic illustration of marker-assisted gene pyramiding of bacterial blight, blast, and sheath blight resistance genes/QTLs in the backgrounds of ASD 16 and ADT 43. RP, recurrent parents (ASD 16 and ADT 43), IRP, improved recurrent parents (ASD 16 and ADT 43 introgressed with *xa5*, *xa13*, and *Xa21* genes); MAS, marker-assisted selection.

Two independent crosses (ASD 16 × IRBB60 and ADT 43 × IRBB60) were made between recipient parents and IRBB60 for targeted gene transfer of *xa5*, *xa13*, and *Xa21*. The hybridity of the F_1_ plants were confirmed by functional/linked PCR-based co-dominant molecular markers of *xa5*, *xa13*, and *Xa21* (Table 1). The heterozygous plants for *xa5*, *xa13*, and *Xa21* were backcrossed with respective recurrent parents (ASD 16 and ADT 43) to generate BC_1_F_1_. The BC_1_F_1_ hybrids were screened for targeted genes (*xa5*, *xa13*, and *Xa21*), and the confirmed plants were assessed with a set of polymorphic SSR markers to recover the maximum percentage of RPG (Sundaram et al., 2008). The solitary plant with targeted genes (*xa5*, *xa13*, and *Xa21*) and maximum recovery of RPG was selected and backcrossed to generate BC_2_F_1_, and this procedure was repeated till BC_3_F_1_. At every backcross, foreground and background selections were carried out to forward the plant with targeted genes and maximum recovery of RPG. Pedigree-based breeding strategy with marker-assisted selection was followed after BC_3_F_1_ to generate BC_3_F_2_ and BC_3_F_3_ populations. In BC_3_F_2_ population, the homozygous plants for targeted genes were identified through foreground selection and self-pollinated to generate BC_3_F_3_ population. The homozygous plants at BC_3_F_3_ were evaluated for resistance against BB as well as for key agronomic traits, and the best lines were pooled to generate three-gene bacterial blight (*xa5*, *xa13*, and *Xa21*) pyramided lines of ASD 16, and ADT 43. The three-gene bacterial blight pyramided lines were crossed with Tetep to introgress blast and sheath blight resistance. The plants showing heterozygous allele for all the targeted genes/QTLs were backcrossed with respective recurrent parent (three-gene bacterial blight pyramided lines of ASD 16 and ADT 43) to generate BC_1_F_1_. The BC_1_F_1_ hybrids were screened with molecular markers for all the targeted traits in the study (Table 1) followed by background selection with polymorphic SSR markers. The “positive” plants with maximum recovery of RPG were again backcrossed with recurrent parents to produce BC_2_F_1_ and BC_3_F_1_. In the BC_3_F_2_ population, foreground selection was carried out to identify the plants carrying all the targeted genes (*xa5* + *xa13* + *Xa21* + *Pi54*) and targeted QTLs (*qSBR7-1* + *qSBR11-1* + *qSBR11-2*) in homozygous condition, and the identified plants were self-pollinated to generate BC_3_F_3_ population. The plants carrying all the targeted traits in homozygous condition at BC_3_F_3_ generation were evaluated for resistance against bacterial blight, blast, and sheath blight diseases under greenhouse conditions and also assessed for key agronomic as well as grain quality traits. The detailed plan of program for marker-assisted gene pyramiding of bacterial blight, blast, and sheath blight resistance genes/QTLs is depicted in Figure 1.

**TABLE 1:** Details of molecular markers used for foreground selection.

**Gene/QTL**	**Marker**	**Sequence (5′–3′)**	**AT (°C)**	**Chr**	**Resistant allele size (bp)**	**Reference**
*Xa21*	pTA248	F-AGACGCGGAAGGGTGGTTTCCCGGA R-AGACGCGGTAATCGAAAGATGAAA	65	11	925	Ronald et al. (1992)
*xa13*	xa13-prom	F-GAGCTCCAGCTCTCCAAATG R-GGCCATGGCTCAGTGTTTAT	59	8	500	Chu et al. (2006)
*xa5*	xa5-1	F-CTCTACCGGAGGTCCACCATTG R-AGGAACAGCAACATTGCAAC	53	5	299	Iyer-Pascuzzi and McCouch (2007)
*Pi54*	Pi54-MAS	F-CAATCTCCAAAGTTTTCAGG R-GCTTCAATCACTGCTAGACC	56	11	216	Ramkumar et al. (2011)
*qSBR7-1*	RM336	F-CTTACAGAGAAACGGCATCG R-GCTGGTTTGTTTCAGGTTCG	55	7	190	Channamallikarjuna et al. (2010)
*qSBR11-1*	RM224	F-ATCGATCGATCTTCACGAGG R-TGCTATAAAAGGCATTCGGG	55	11	130	
*qSBR11-2*	RM209	F-ATATGAGTTGCTGTCGTGCG R-CAACTTGCATCCTCCCCTCC	55	11	150	

### DNA Extraction and PCR Amplification

The DNA extraction for PCR amplification was carried out by the CTAB method (Varghese et al., 1997). The PCR protocol for marker-assisted selection of targeted genes/QTLs was followed according to the earlier reports (Chu et al., 2006; Iyer-Pascuzzi and McCouch, 2007; Sundaram et al., 2008; Channamallikarjuna et al., 2010; Ramkumar et al., 2011; Table 1). Ten microliters of PCR reaction mixture contains 4 μl of DreamTaq green 2× PCR master mix (Thermo scientific, United States), 4 μl of water, 50 ng of template DNA, and 30 ng each of forward and reverse primers. To find the specific allelic pattern of *xa*5 allele, 5–10 μl of PCR product is digested with *Bsr*I (5 units of enzyme) at 65°C for 4 h with 2 μl of 10×PCR buffer (Iyer-Pascuzzi and McCouch, 2007). The amplified PCR products were separated by 2.5% agarose gel stained with ethidium bromide and visualized on UV light in gel documentation system (Bio-Rad Laboratories., United States). Background selection was carried out with a set of 463 SSR markers^1^; 69 and 68 markers were found to be polymorphic for ADT 43 and ASD 16 cross combinations, respectively (Supplementary Table S1), with a wide coverage of all the 12 chromosomes (five to six polymorphic markers per chromosome). Additional polymorphic markers, i.e., 10 polymorphic markers were employed on chromosome number 11, which carries *Xa21*, *Pi54*, *qSBR11-1*, and *qSBR11-2* to minimize the linkage drag.

### Bioassays Against Bacterial Blight, Blast, and Sheath Blight Diseases

#### Bacterial Blight

The selected IPLs (improved pyramided lines) and parents (IRBB60 as resistant check and ASD 16 and ADT 43 as susceptible checks) were tested for resistance against the DX-027 of *Xoo* isolate under greenhouse conditions. In addition to the selected IPLs, single- and two-gene BB pyramided lines were also evaluated to check the effectiveness of three-gene BB pyramided lines. Three replications were maintained with 30 plants per replication. The top leaves were clipped off, and bacterial suspension was inoculated with a density of 10^9^ cells/ml by clip inoculation method at maximum tillering stage (Kauffman et al., 1973). Eight leaves per plant were inoculated, and mean lesion length was taken on six leaves to measure the accurate disease reaction. Symptoms were measured 21 days post-inoculation, and observations were recorded based on visual score and lesion length (LL). The plants with an average lesion length of <5 cm were considered as resistant, and those with >5 cm were considered as susceptible (International Rice Research Institute [IRRI], 2002).

#### Blast

The IPLs and parents (Tetep as resistant check; ASD 16 and ADT 43 as susceptible checks) were evaluated for leaf blast resistance against IS (KUL)-6, a virulent local isolate of *M. oryzae*. Five-week-old seedlings were individually transplanted into mud pots (19 cm × 22 cm × 22 cm) with three replications and inoculated with *M. oryzae* IS (KUL)-6 isolate at a spore density of 5 × 10^5^ spores/ml. Disease reaction was recorded 9 days post-inoculation and evaluated based on blast lesion type (BLT) according to the 0–9 scale of SES (Standard Evaluation System) (International Rice Research Institute [IRRI], 2002). The plants with a score of 0–3 were rated as resistant and those with more than a score of 4 were rated as susceptible.

#### Sheath Blight

A pure culture of *R. solani* collected from the Tamil Nadu Rice Research Institute (TRRI), Aduthurai, Tamil Nadu, India, was used for testing the resistance of IPLs and parents against sheath blight. The pathogen was multiplied on sterilized shoot bits of water sedge, *Typha angustata*. Infected *Typha* shoot bits with mycelium and sclerotia were used as source of inoculum and placed carefully between the tillers of rice hills with the help of forceps at 80 days after sowing. The inoculated portion was covered with wet cotton and aluminum foil to avoid the moisture loss in the inoculated portion. The observations were recorded 25 days post-inoculation on randomly selected plants consisting of three infected tillers in each of three replications. Disease reaction was measured based on RLH% (relative lesion height), and scoring (0–9) was given as per SES (International Rice Research Institute [IRRI], 2002) [0 (immune), 1–20% (resistant), 21–30% (moderately resistant), 31–45% (moderately susceptible), 46–65% (susceptible), and >65% (highly susceptible)].

### Characterization of Agro-Morphological and Quality Traits

The 30-day old seedlings of recurrent parents and selected IPLs were transplanted to an experimental plot at the Agricultural College and Research Institute, Tamil Nadu Agricultural University, Madurai, India, with a spacing of 15 cm × 20 cm. The experimental plot was arranged in a randomized block design (RBD) with four blocks, and three replications were maintained in each block. Standard agronomic practices were followed as prescribed by TNAU, Coimbatore, India^2^, to raise the healthy crop. Observations were recorded on five plants in each line for key agronomic traits *viz*., days to 50% flowering (DFF), plant height (PH) (cm), number of productive tillers per plant (NPT), panicle length (PL) (cm), number of grains per panicle (NGP), flag leaf length (FL) (cm), flag leaf width (FW) (cm), 1,000-grain weight (1,000-GW) (g), grain-L/B ratio (cm), and single plant yield (SPY) (g). In addition, the quality traits *viz.*, hulling percentage (HP%), milling percentage (MP%), head rice recovery (HRR%), kernel length (KL) (mm), kernel breadth (KB) (mm), kernel length breadth ratio (KLBR), milled rice length (MRL) (mm), milled rice breadth (MRB) (mm), kernel length after cooking (KLAC) (mm), kernel breadth after cooking (KBAC) (mm), and linear elongation ratio (LER) were analyzed in the homozygous improved pyramided lines.

### Statistical Analysis

Statistical analysis was done with SPSS software to complement the ANOVA (analysis of variance) to determine the significant variation among the improved pyramided lines. The coefficient of genetic distance among the selected pyramided lines and parents was calculated based on 10 morphological characters and used for generating the dendrogram using the “R” software (R Core Team, 2013).

## Results

### Marker-Aided Pyramiding of *Xa21*, *xa13*, and *xa5* in the Backgrounds of ASD 16 and ADT 43

The hybrids (F_1_) from the crosses of ASD 16 × IRBB60 and ADT 43 × IRBB60 were analyzed with functional/linked molecular markers of *xa5*, *xa13*, and *Xa21*, i.e., *xa5*-1, *xa*-13 prom and pTA 248, respectively. A total of 12/138 plants in ASD 16 × IRBB60 cross combination and 15/157 plants in ADT 43 × IRBB60 cross combination were found to be “positive” for all the targeted genes (*xa5*, *xa13*, and *Xa21*). These positive plants were backcrossed with respective recurrent parents (ASD 16 and ADT 43) to generate the BC_1_F_1_ population. We have found that 9/134 plants in ASD 16 background and 13/150 plants in ADT 43 background were heterozygous for *xa5*, *xa13*, and *Xa21*, and these plants were screened with polymorphic SSR markers to assess the recovery of RPG. Background assay revealed that three plants in ASD 16 combination *viz*., IL-1-2-2, IL-1-5-6, and IL-1-8-18 with an RPG% recovery of 75.60, 76.82, and 76.56, respectively, and two plants in ADT 43 combination *viz*., IL-2-1-33 and IL-2-2-53 with a recovery of 77.08% and 76.04% were identified (Supplementary Table S2). These plants were backcrossed alone with respective recurrent parents to produce BC_2_F_1_. In BC_2_F_1_, 10/154 plants in ASD 16 background and 12/166 plants in ADT 43 background were found to be heterozygous for the targeted genes. In these BC_2_F_1_ populations, we have identified two plants in ASD 16 combination and three plants in ADT 43 combination with background genome recovery ranging from 87.5 to 89.02% (Supplementary Table S2), and these plants were backcrossed to produce BC_3_F_1_. In BC_3_F_1_, we have found that 11/92 in ASD 16 background and 17/110 in ADT 43 background were shown to be triple heterozygous, and background assay indicates that three plants with RPG recovery ranged from 93.85 to 94.96% in ASD 16 combination, and two plants with a recovery of 94.26% and 94.04% were observed in ADT 43 combination (Supplementary Table S2). Self-pollination was carried out in the selected plants to generate BC_3_F_2_ followed by BC_3_F_3_. Homozygous plants for *xa5*, *xa13*, and *Xa21* with key agro-morphological traits were identified through genotypic and phenotypic assays, and the best lines (six lines in ASD 16 combination and nine lines in the ADT 43 combination) were constituted to generate triple-gene (*xa5*, *xa13*, and *Xa21*) pyramided lines of ASD 16 and ADT 43 for bacterial blight resistance.

### Combining Multiple Disease (BB, Blast, and Sheath Blight) Resistant Genes/QTLs in the Backgrounds of ASD 16, and ADT 43

**FIGURE 2:**
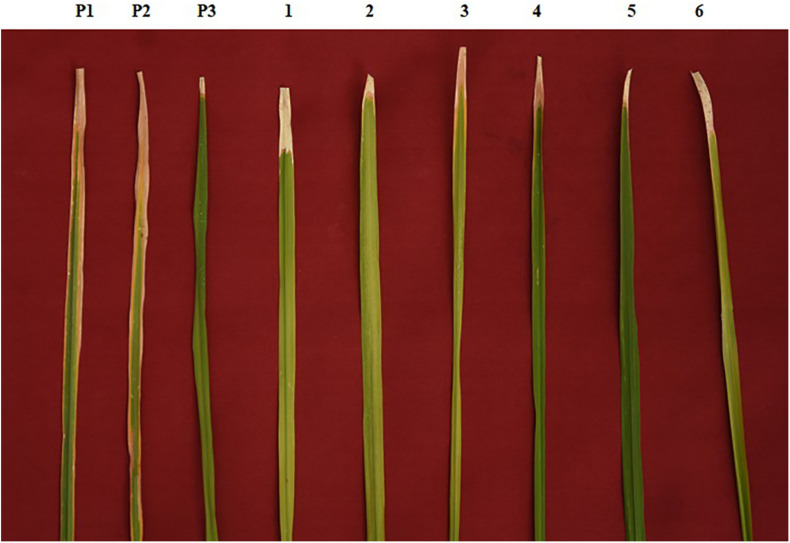
Phenotypic screening of improved pyramided lines against DX-027 isolate of bacterial blight (BB). P1 – ASD 16, P2 – ADT 43, P3 – IRBB60; serially numbered 1, 2, 3, 4, 5, and 6 lines indicate improved pyramided lines of recurrent parents harboring *xa5*, *xa13*, and *Xa21* genes.

**FIGURE 3:**
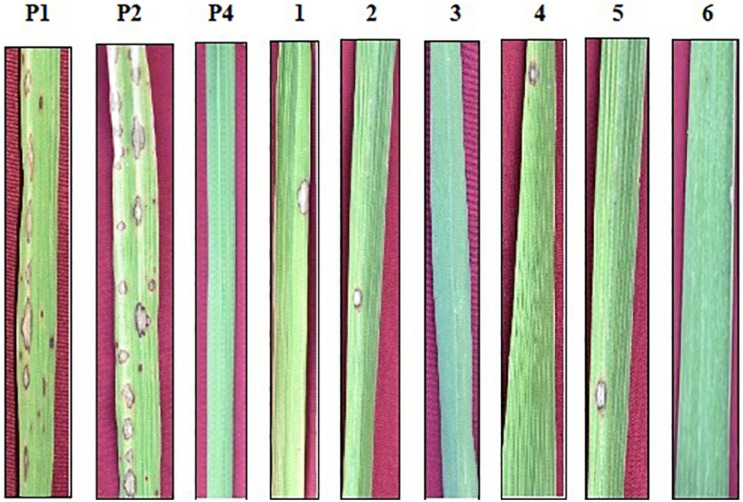
Phenotypic screening of improved pyramided lines against IS (KUL)-6 isolate of *Magnaporthe oryzae* for leaf blast. P1 – ASD 16, P2 – ADT 43, P4 – Tetep; serially numbered 1, 2, 3, 4, 5, and 6 lines indicate improved pyramided lines of recurrent parents harboring the *Pi54* gene.

**FIGURE 4:**
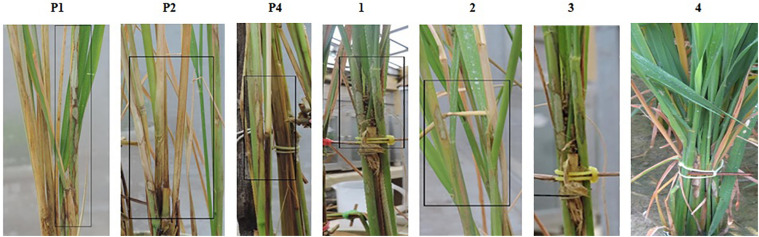
Phenotypic screening of improved pyramided lines against sheath blight resistance. P1 – ASD 16, P2 – ADT 43, P4 – Tetep; serially numbered 1, 2, 3, and 4 lines indicate improved pyramided lines harboring *qSBR7-1*, *qSBR11-1*, and *qSBR11-2* QTLs.

**FIGURE 5:**
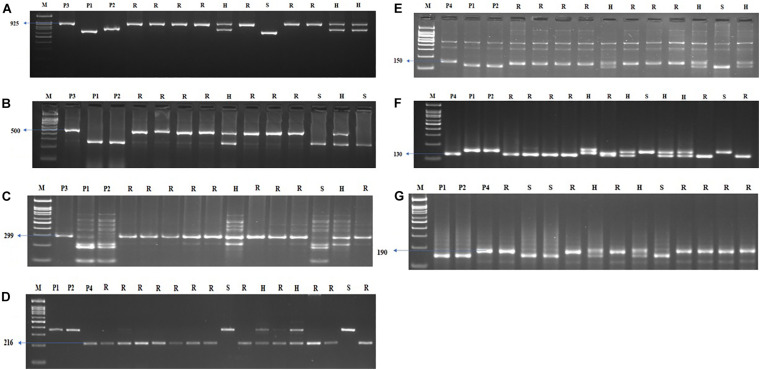
Agarose gel electrophoresis images illustrating the presence of **(A)** *Xa21*, **(B)** *xa13*, **(C)** *xa5*, **(D)** *Pi54*, **(E)** *qSBR11-2*, **(F)** *qSBR11-I*, and **(G)** *qSBR7-1* alleles. P1 – ASD 16, P2 – ADT 43, P3 – IRBB60, P4 – Tetep, M – 100-bp ladder, R, resistant; H, heterozygote; S, susceptible.

The three-gene bacterial blight (*xa5*, *xa13*, and *Xa21*) pyramided lines of ASD 16 and ADT 43 were used as recipient parents for the targeted introgression of blast (*Pi54*) and sheath blight QTLs (*qSBR7-1*, *qSBR11-1*, and *qSBR11-2*) from the Tetep. The bacterial blight pyramided lines of ASD 16 and ADT 43 harboring *xa5*, *xa13*, and *Xa21* were crossed with Tetep, and the F_1_ plants were analyzed for all the targeted traits (Table 1). The hybrids with *xa5* + *xa13* + *Xa21* + *Pi54* + *qSBR7-1* + *qSBR11-1* + *qSBR11-2* in heterozygous condition were selected and backcrossed with ASD 16 and ADT 43 introgressions with the three-gene bacterial blight genes (*xa5*, *xa13*, and *Xa21*) to recover the original RPG. Up to BC_3_F_1_, all the foreground and background selections were made similar to pyramiding of bacterial blight-resistant genes, as explained above. Foreground selection of BC_3_F_1_ hybrids revealed five plants in ASD 16 background and seven plants in ADT 43 background possessing all the targeted genes and QTLs (*xa5* + *xa13* + *Xa21* + *Pi54* + *qSBR7-1* + *qSBR11-1* + *qSBR11-2*) in heterozygous condition. Background selection of these positive plants revealed two plants with RPG recovery ranging from 93.88 to 94.92% in both combinations (ASD 16, and ADT 43) (Supplementary Table S2). Selfing was done in “positive” BC_3_F_1_ hybrids with high RPG recovery to generate BC_3_F_2_. In BC_3_F_2_ generation, 11 plants in the ASD 16 background and 14 plants in the ADT 43 background were homozygous for targeted genes (*xa5* + *xa13* + *Xa21* + *Pi54*) and targeted QTLs (*qSBR7-1* + *qSBR11-1* + *qSBR11-2*), and these were selfed to produce BC_3_F_3_. These homozygous-improved gene pyramided lines at BC_3_F_3_ generation were evaluated for physical expression of resistance against bacterial blight, blast, and sheath blight pathogens under greenhouse conditions. Out of 1,500 plants screened in both the crosses at field conditions, we found nine lines in ASD 16 background and 15 lines in ADT 43 background performing on par with the recurrent parents and, moreover, exhibiting a high level of resistance to all the three major diseases (Tables 2,3 and Figures 2–5).

**TABLE 2:** Phenotypic reaction and disease score of improved pyramided lines against bacterial blight (BB), blast, and sheath blight diseases.

**Genotype**	**Bacterial blight**		**Blast**	**Sheath blight**	
	**LL (cm)**	**Score**	**Score**	**RLH (%)**	**Score**
ACM 18243	3.15 ± 0.56	0	2	44	5
ACM 18244	3.28 ± 0.67	0	1	28	3
ACM 18242	3.17 ± 1.43	1	1	39	5
ACM 18245	3.35 ± 1.02	1	3	30	3
ACM 18249	3.67 ± 0.62	0	1	37	5
ACM 18012	4.03 ± 0.34	1	2	43	5
ACM 18014	3.87 ± 0.34	0	1	38	5
ACM 18015	3.36 ± 0.70	0	0	29	3
ACM 18023	3.33 ± 0.78	1	1	35	5
ACM 18020	4.21 ± 0.49	1	2	30	3
IRBB60	3.10 ± 0.45	0	–	–	–
Tetep	–	–	0	28	3
ASD 16	15.12 ± 1.46	9	9	78	9
ADT 43	16.88 ± 0.56	9	9	72	9

*LL, lesion length;RLH (%), relative lesion height expressed in percentage;BB – LL of <5 cm is considered as resistant, and LL > 5 cm is considered as susceptible. Blast – 0–1 (highly resistant), 2–3 (resistant), 4 (moderately resistant), 5–6 (moderately susceptible), 7 (susceptible), and 8–9 (highly susceptible). Sheath blight – 0 (Immune), 1 (resistant), 3 (moderately resistant), 5 (moderately susceptible), 7 (susceptible), and 9 (highly susceptible).*

**TABLE 3:** Agro-morphological characters of selected improved pyramided lines at BC_3_F_3_ generation.

**Genotype**	**Days to 50% flowering (DFF)**	**Plant height (PH) (cm)**	**Flag leaf length (FL) (cm)**	**Flag leaf width (FW) (cm)**	**Number of productive tillers per plant (NPT)**	**Panicle length (PL) (cm)**	**Number of grains per panicle (NGP)**	**1,000-Grain weight (1,000-GW) (g)**	**L/B ratio**	**Single plant yield (SPY) (g)**
ACM 18012	85	88.3 ± 1.2	32.5 ± 0.6	1.0 ± 0.01	18 ± 2.1	24.2 ± 0.4	199 ± 4.5	15.21 ± 0.2	3.71 ± 0.02	27.11 ± 0.4
ACM 18242	64	87.4 ± 1.4	29.3 ± 0.4	1.3 ± 0.02	16 ± 1.4	26.5 ± 0.3	205 ± 9.7	22.9 ± 0.3	2.32 ± 0.04	30.13 ± 0.3
ACM 18243	82	89.1 ± 2.1	28.4 ± 0.4	1.2 ± 0.01	20 ± 3.2	23.4 ± 0.6	207 ± 8.1	22.89 ± 0.5	2.43 ± 0.01	30.19 ± 0.4
ACM 18244	89	86.4 ± 1.1	28.8 ± 0.5	1.2 ± 0.01	17 ± 2.1	20.6 ± 0.2	202 ± 9.4	24.15 ± 0.1	2.59 ± 0.02	30.24 ± 0.4
ACM 18013	78	87.6 ± 1.2	33.1 ± 0.4	1.0 ± 0.02	14 ± 3.2	19.3 ± 1.8	194 ± 12.6	16.32 ± 0.6	3.68 ± 0.05	28.06 ± 0.7
ACM 18014	81	86.5 ± 1.8	32.5 ± 0.3	1.0 ± 0.03	16 ± 1.4	20.5 ± 1.2	188 ± 10.3	15.94 ± 0.4	3.61 ± 0.03	27.62 ± 0.8
ACM 18245	79	88.3 ± 1.7	29.4 ± 0.4	1.3 ± 0.01	14 ± 2.4	27.8 ± 0.3	212 ± 11.2	23.61 ± 0.4	2.62 ± 0.02	29.65 ± 0.4
ACM 18246	65	83.8 ± 2.3	28.7 ± 0.1	1.2 ± 0.02	16 ± 2.1	24.3 ± 1.1	201 ± 6.8	22.78 ± 0.5	2.32 ± 0.01	27.19 ± 1.1
ACM 18247	88	78.8 ± 3.4	29.3 ± 0.4	1.2 ± 0.03	14 ± 3.2	21.2 ± 0.6	213 ± 14.1	22.19 ± 0.1	2.18 ± 0.01	29.89 ± 0.3
ACM 18015	83	79.7 ± 3.8	33.2 ± 0.3	1.0 ± 0.01	15 ± 1.5	20.8 ± 1.4	198 ± 12.2	15.29 ± 0.4	3.78 ± 0.05	28.18 ± 0.2
ACM 18016	84	85.4 ± 2.4	32.8 ± 0.6	0.9 ± 0.01	15 ± 2.1	21.5 ± 0.5	198 ± 13.4	15.23 ± 0.6	3.91 ± 0.03	27.95 ± 1.2
ACM 18017	81	88.3 ± 1.3	33.1 ± 0.2	0.9 ± 0.02	16 ± 2.3	23.8 ± 0.8	192 ± 11.9	16.65 ± 1.2	3.89 ± 0.08	28.24 ± 0.5
ACM 18248	85	86.2 ± 1.7	28.6 ± 0.5	1.2 ± 0.02	14 ± 2.8	23.7 ± 0.5	188 ± 16.6	24.08 ± 0.6	2.32 ± 0.05	29.85 ± 0.8
ACM 18018	85	81.5 ± 2.5	33.2 ± 0.2	0.9 ± 0.01	18 ± 1.2	18.3 ± 1.8	185 ± 6.5	15.27 ± 0.4	3.81 ± 0.04	27.49 ± 1.1
ACM 18249	85	81.6 ± 2.6	29.1 ± 0.2	1.1 ± 0.06	16 ± 1.4	24.1 ± 1.1	194 ± 8.5	22.34 ± 0.1	2.24 ± 0.04	29.71 ± 0.4
ACM 18250	79	79.7 ± 3.9	29.2 ± 0.5	1.2 ± 0.03	16 ± 2.1	23.4 ± 0.7	197 ± 8.1	22.56 ± 0.8	2.14 ± 0.06	27.15 ± 1.1
ACM 18019	83	89.4 ± 0.8	32.8 ± 0.3	0.9 ± 0.02	14 ± 2.4	23.8 ± 0.4	195 ± 12.9	15.15 ± 1.1	3.68 ± 0.04	28.23 ± 0.7
ACM 18020	81	81.3 ± 3.1	33.1 ± 0.1	1.0 ± 0.01	17 ± 3.3	25.9 ± 0.3	196 ± 4.8	15.87 ± 1.3	3.72 ± 0.06	27.48 ± 0.5
ACM 18021	82	85.3 ± 1.1	32.4 ± 0.2	1.0 ± 0.01	17 ± 1.2	24.2 ± 0.4	195 ± 3.4	15.52 ± 1.1	3.69 ± 0.02	26.2 ± 1.1
ACM 18022	83	84.7 ± 1.9	33.2 ± 0.1	0.9 ± 0.01	16 ± 1.6	24.6 ± 0.2	182 ± 4.7	15.23 ± 0.4	3.69 ± 0.05	25.8 ± 0.4
ACM 18023	78	82.5 ± 2.3	32.7 ± 0.3	0.9 ± 0.03	17 ± 1.2	25.1 ± 0.3	189 ± 2.1	15.29 ± 0.8	3.70 ± 0.02	26.4 ± 0.2
ASD 16	86	84.4 ± 3.1	29.3 ± 0.4	1.2 ± 0.05	19 ± 2.6	22.5 ± 0.6	201 ± 11.2	23.2 ± 0.5	2.08 ± 0.01	29.2 ± 0.2
ADT 43	85	85.2 ± 2.4	32.7 ± 0.4	1.0 ± 0.03	18 ± 1.3	24.3 ± 0.8	191 ± 14.3	15.6 ± 0.4	3.69 ± 0.07	27.6 ± 0.4
IRBB60	91	87.7 ± 4.8	31.1 ± 0.4	1.1 ± 0.02	15 ± 2.5	22.83 ± 2.5	148 ± 7.8	18.76 ± 0.6	3.34 ± 0.04	25.32 ± 0.3
Tetep	97	123.2 ± 5.8	35.5 ± 0.3	0.85 ± 0.05	17 ± 2.2	27.13 ± 3.1	153 ± 19.7	19.15 ± 1.2	3.43 ± 0.05	26.15 ± 0.9
**SE**	1.199	0.646	0.392	0.0282	0.312	0.471	1.594	0.762	0.142	0.296
**CD (5%)**	1.74	1.62	0.6	0.05	1.34	0.75	3.81	0.54	0.02	0.36

### Evaluation of Improved Pyramided Lines for Resistance to Bacterial Blight, Blast, and Sheath Blight Diseases

#### Bioassay for BB Resistance

The resistant parent, IRBB60, possessing *xa5*, *xa13*, and *Xa21* exhibited a mean lesion length of 3.10 ± 0.45 with a disease reaction score of 0–1 (highly resistant) against the isolate of DX-027. Similarly, the identified homozygous IPLs were also expressed, and the similar mean lesion length ranged from 3.15 ± 0.56 to 4.21 ± 0.49 with the same disease reaction score as the resistant parent. The two-gene pyramided lines (*xa5* + *xa13*, *xa5* + *Xa21*, and *xa13* + *Xa21*) were shown a mean lesion length of 4.58 ± 1.15 with a score of 1–2 (resistant). Evaluation of single-gene pyramided lines showed that *Xa21* pyramided lines expressed a mean lesion length of 4.93 ± 0.22, *xa13* lines with 6.45 ± 0.79, and *xa5* lines with 6.85 ± 0.64 lesion lengths. This indicates that the lines that harbor the *Xa21* component exhibited a high level of resistance than the other components in the disease reaction (data not shown), while the recurrent parents (ASD 16 and ADT 43) have shown an average lesion length of more than 15 cm with a disease reaction score of 9 (highly susceptible) for the same isolate of BB (Table 2 and Figure 2).

#### Bioassay for Blast and Sheath Blight Resistance

The resistant check, Tetep, possessing the *Pi54* gene, exhibited a high level of resistance to leaf blast with no lesions observed on the leaf with a disease score of 0–1 (highly resistant) against the isolate of IS (KUL)-6. Similarly, the selected IPLs harboring the *Pi54* gene displayed small pin-point size brown specks to slightly elongated necrotic patches with a sporulating center with a disease score of 1–3 (highly resistant to resistant). While, the recurrent parents (ASD 16 and ADT 43) displayed spindle-shaped lesions with brown margin with a disease reaction score of 9 (highly susceptible) (Table 2 and Figure 3). With regard to the sheath blight, Tetep possessing *qSBR* QTLs (*qSBR7-1*, *qSBR11-1*, and *qSBR11-2*) exhibited an RLH up to 28% with a disease score of 3 (moderately resistant). The susceptible checks, both ASD 16 and ADT 43, expressed an RLH of 78% and 71%, respectively, with disease score of 9 (highly susceptible). While the selected IPLs possessing *qSBR7-1*, *qSBR11-1*, and *qSBR11-2* have expressed an RLH ranging from 28 to 45% with a disease score of 3 to 5 (moderately resistant to moderately susceptible) (Table 2 and Figure 4).

### Evaluation of Improved Pyramided Lines for Agro-Morphological and Quality Traits

All the selected improved pyramided lines (nine lines in ASD 16 background and 15 lines in ADT 43 background) have similar agro-morphological and quality traits as recurrent parents. Some promising lines have shown superior agro-morphological and quality traits and, moreover harboring BB, blast, and sheath blight resistance. Significant differences for plant height were observed among the few improved pyramided lines (BC_3_F_3_ generation), which were shown as taller than recurrent parents *viz.*, ACM 18012, ACM 18245, ACM 18242, ACM 18243, ACM 18244, ACM 18013, and ACM 18014 (Table 3). The DFF for recurrent parents (ASD 16 and ADT 43) and donor parents (IRBB60 and Tetep) were 85 and 97 days, respectively. The selected improved pyramided lines at BC_3_F_3_ generation harboring *xa5* + *xa13* + *Xa21* + *Pi54* + *qSBR7-1* + *qSBR11-1* + *qSBR11-2* also showed a similar range for DFF, i.e., 78–95 days. Two lines in the background of ASD 16 *viz*., ACM 18242 and ACM 18246 were significantly flowered ∼13–18 days earlier than its respective recurrent parent, ASD 16 (Table 3). The mean values of recurrent parents for the number of grains per panicle ranged from 191 ± 14.3 to 201 ± 11.2. Several improved pyramided lines *viz*., ACM 18012, ACM 18245, ACM 18242, ACM 18243, ACM 18017, ACM 18016, ACM 18247, and ACM 18015 have shown significantly higher number of grains per panicle than both recurrent parents (Table 3). The mean 1,000-grain weight (g) of recurrent parents ranged from 15.6 ± 0.4 to 23.2 ± 0.5 for ADT 43 and ASD 16, respectively. The selected improved pyramided lines, ACM 18245, ACM 18244, ACM 18013, ACM 18016, ACM 18017, and ACM 18050 have shown significantly higher 1,000-grain weight than both the recurrent parents ASD 16 and ADT 43 (Table 3). The HRR% of recurrent parents ranged from 59.87 to 62.14 for ADT 43 and ASD 16, respectively, while the selected homozygous lines HRR% are also on par with the recurrent parents and some of the lines have recovered more HRR% than both the recurrent parents (Supplementary Table S3). Some of the promising lines, ACM 18012, ACM 18014, and ACM 18015 in the background of ADT 43 and ACM 18244, ACM 18245, and ACM 18249 in the background of ASD 16 have shown superior quality traits than both the recurrent parents (Supplementary Table S3).

### Cluster Analysis

**FIGURE 6:**
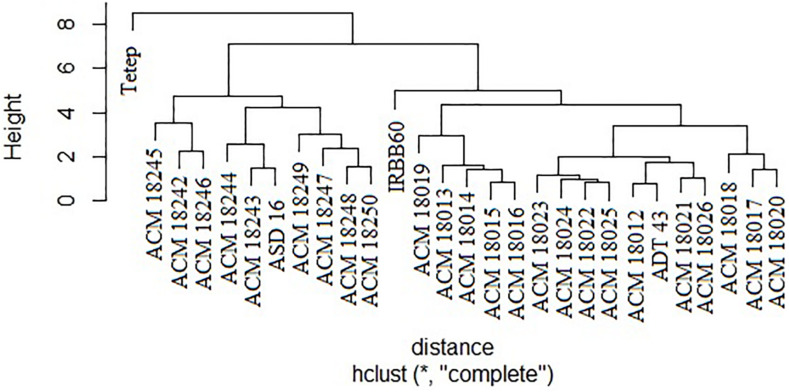
Clustering of 24 selected improved pyramided lines based on 10 morphological characters. ^∗^Euclidean distance.

The coefficient of genetic distance on 10 morphological traits of 24 pyramided lines and four parents revealed that all the pyramided lines were very similar with their respective recurrent parent. It was observed that two solitary clusters were formed, one with Tetep alone and another with improved pyramided lines, IRBB60 and recurrent parents (ASD 16, and ADT 43). Cluster II was subdivided into two clusters, i.e., one subcluster with ASD 16 and its respective pyramided lines, another subcluster with IRBB60, ADT 43 parent, and its respective pyramided lines. Obviously, all the improved pyramided lines were being clubbed into their respective recurrent parent cluster (Figure 6).

## Discussion

More than half of the population of the globe is consuming rice to meet their dietary requirements. The demand for rice production is increasing, and many studies have reported that global rice production need to be doubled to meet the demands of the growing population (Ray et al., 2013). Besides the growing population, many biotic and abiotic stresses are affecting both yield and quality of rice crop. To address these constraints and to increase rice production, development of high-yielding cultivars enriched with resistant genes will enhance the yield as well as host-plant resistance. Conventional backcross method is the primary approach to develop resistant cultivars for single gene resistance, but phenotypic selection is a difficult and time-consuming process when multiple genes are involved in disease resistance (Crossa et al., 2017). The use of marker-assisted selection with stringent phenotypic selection enhances the efficiency and precision of breeding program for developing multiple disease-resistant varieties.

Marker-assisted backcross breeding (MABB) is a fastidious method for introgression of two or more targeted genes in the elite cultivars for improving the deficient trait. The prime aim of MABB is to transfer the targeted genes into the background of elite cultivars and to recover the RP genome as quickly as possible with a limited number of backcrosses. MABB also focuses on gradual reduction of donor parent genome as much as possible to avoid the undesirable effects on agronomical, yield, and quality traits. The recurrent parents (ASD 16 and ADT 43) in this study are the popular and high-yielding cultivars of South India. Bacterial blight, blast, and sheath blight diseases are the major diseases of South India causing huge losses to the crop.

Many studies have focused on developing resistance to one or two diseases, as developing resistance to more than two diseases is a complex and time-consuming process. There are a few reports on developing multiple disease-resistant cultivars. Singh et al. (2012) introgressed blast and sheath blight resistance genes/QTLs in the background of Improved Pusa Basmati 1, harboring *xa13*, and *Xa21* genes. Das and Rao (2015) introgressed blast (*Pi2* and *Pi9*), submergence (*Sub1*), gall midge (*Gm1*, *Gm4*), and salinity (*Saltol1*) genes/QTLs in the background of Improved Lalat harboring *Xa4*, *xa5*, *xa13*, and *Xa21*. Arunakumari et al. (2016) has introgressed bacterial blight and blast resistance genes into the popular cultivar MTU 1010. As a part of sustainable management, we have planned and executed the introgression of BB (*xa5*, *xa13*, and *Xa21*), blast (*Pi54*), and sheath blight (*qSBR7-1*, *qSBR11-1*, and *qSBR11-2*) resistance genes/QTLs in the backgrounds of ASD 16 and ADT 43 for achieving multiple disease resistance and to offer IPL to farmers.

In this study, background screening with polymorphic SSR markers was used to recover the maximum percentage of RPG in early generations. At BC_3_F_1_, we have achieved RPG recovery in both ASD 16 and ADT 43 backgrounds. The background recovery rate in this study was at par with the theoretical recovery rate. We have observed inheritance of some unfavorable characters (plant height and grain qualities) along with favorable resistant traits while introgressing genes/QTLs from Tetep. Nevertheless, we have identified superior segregates with minimal residual effect from the Tetep genome by assessing greater population size (1,500 plants in both crosses). Sundaram et al. (2008), reported that introgression of *Xa21*, *xa13*, and *xa5* genes from SS1113 exercises a “pull” through inheritance of undesirable loci from a donor segment. Singh et al. (2015) introgressed *qSBR7-1*, *qSBR11-1*, and *qSBR11-2* into Pusa 6B, but they have not observed the inheritance of undesirable loci from Tetep. The observed undesirable effects of Tetep on the agronomic characters might be due to the linkage drag or environmental influence on agronomic characters.

The agronomic performance of 24 selected improved pyramided lines of both ASD 16 and ADT 43 backgrounds at BC_3_F_3_ generation revealed that most of the agro-morphological traits were on par with their recurrent parents (ASD 16 and ADT 43) and also showed durable resistance to BB, blast, and sheath blight diseases. The higher yield and superior quality traits of improved pyramided lines were probably due to the inheritance of yield contributing traits from the recurrent parents. The yield, agro-morphological, and quality traits were normally controlled by polygenes, and these are distributed throughout the genome. Employment of a greater number of background markers accelerated the recovery of RPG in the early generations. Cluster analysis of selected pyramided lines and parents based on 10 morphological characters revealed that all the selected pyramided lines were clustered in their recurrent parent’s cluster. This is due to the similar morphological characters of pyramided lines with their recurrent parent’s. Similar results were also obtained by Pradhan et al. (2015) and Hsu et al. (2020), while introgressing BB resistance genes, which supports the present study results.

The homozygous improved pyramided lines (BC_3_F_3_ generation) harboring *xa5* + *xa13* + *Xa21* + *Pi54* + *qSBR7-1* + *qSBR11-1* + *qSBR11-2* were assessed for physical resistance under greenhouse conditions. The results of bioassays suggest that pyramiding three BB-resistant genes exhibited higher resistance levels than the lines with one or two genes. In addition, the present study results also reveal that the gene combination with *Xa21* component expressed a shorter lesion length than the remaining gene combinations. These results were consistent with the results of previous studies (Peng et al., 2015; Pradhan et al., 2015; Ramalingam et al., 2017; Yugander et al., 2018; Hsu et al., 2020). The improved pyramided lines harboring the *Pi54* gene and ShB-resistant QTLs have expressed a similar disease reaction as donor parent, Tetep. This is due to the transfer of resistant alleles from the donor parent, which was confirmed by functional/linked molecular markers as well as phenotypic screening methods.

The field evaluation of improved pyramided lines at BC_3_F_3_ generation demonstrated that the candidate lines of both recurrent parents had equivalent expression of yield, agro-morphological, and quality traits and, more importantly, with pyramided genes for BB, blast, and sheath blight. The higher levels of resistance to multiple diseases, without any yield penalty, is an integrated approach of genotypic and phenotypic selection methods. Developing broad-spectrum resistance to multiple diseases is a challenging task due to rich diversity of agro-climatic conditions in India along with the existence of genetically distinct virulent strains of different plant pathogens. Pyramiding of multiple or effective resistant genes/QTLs for different biotic stresses can contribute broad-spectrum and durable resistance to multiple diseases to the rice regions in India. The present study results prove that MABB is an effective tool for pyramiding major genes/QTLs to obtain improved plant lines in a quick time frame.

Introgression of BB resistance genes from IRBB60, blast and sheath blight resistance genes/QTLs from Tetep into the commercial cultivars is a significant achievement for obtaining durable resistance to multiple diseases. Introgression of identified effective resistance genes from the wild relatives or landraces into the commercial cultivars gradually improves the host-plant resistance to different biotic and abiotic stresses for attaining food and nutritional security. In conclusion, we have introgressed *xa5*, *xa13*, *Xa21*, *Pi54*, and *qSBR* QTLs (*qSBR7-1*, *qSBR11-1*, and *qSBR11-2*) in the backgrounds of ASD 16 and ADT 43 to improve host-plant resistance to BB, blast, and sheath blight diseases. The improved pyramided lines can be further tested in multilocational trails and could be released as improved variety or used as a potential donor in hybridization programs for developing multiple disease-resistant cultivars in rice.

## Data Availability Statement

The original contributions presented in the study are included in the article/Supplementary Material, further inquiries can be directed to the corresponding author.

## Author Contributions

JR designed the experiment. ChR, PS, RS, VV, and TC were involved in the experiments. JR, ChR, RS, and PS developed the improved pyramided lines. SV and MA were involved in a part of the experiment. JR, ChR, VV, and AR were involved in the screening of improved pyramided lines. JR, SA, and CV were involved in the revision of the final version of the manuscript. JR and all the authors prepared and approved the final version of the manuscript.

## Conflict of Interest

The authors declare that the research was conducted in the absence of any commercial or financial relationships that could be construed as a potential conflict of interest.

## Footnotes

1 https://archive.gramene.org/

2 http://agritech.tnau.ac.in/
