# Supplementary material for: Gene Pyramiding for Achieving Enhanced Resistance to Bacterial Blight, Blast, and Sheath Blight Diseases in Rice
Source: Front Plant Sci. 2020 Nov 19;11:591457. doi: 10.3389/fpls.2020.591457 (PMC7711134; doi:10.3389/fpls.2020.591457)
Supplement: Supplementary file 1 [file Data_Sheet_1.docx]

**Table S1 |** Details of polymorphic SSR markers used for background screening

| **Chromosome number** | **Polymorphic markers of ADT 43, IRBB60 and Tetep** | **Polymorphic markers of ASD 16, IRBB60 and Tetep** |
| --- | --- | --- |
| 1 | RM84, RM8051, RM493, RM488, RM490, RM3412 | RM443, RM443, RM8077, RM473, RM3412, RM6515 |
| 2 | RM154, RM555, RM3316, RM3263, RM109 | RM6165, RM3263, RM109, RM262, RM279 |
| 3 | RM520, RM6283, RM1002, RM545, RM15080 | RM1002, RM251, RM545, RM6283, RM15080 |
| 4 | RM574, RM252, RM3843, RM1153, RM307 | RM252, RM1153, RM307, RM3843 |
| 5 | RM159, RM163, RM538, RM13, RM430, RM4837 | RM267, RM5140, RM274, RM440, RM153, RM289 |
| 6 | RM585, RM276, RM3183, RM461, RM469, RM435 | RM469, RM461, RM400, RM435, RM3183, RM276 |
| 7 | RM6697, RM432, RM234, RM21976, RM445 | RM473A, RM445, RM18, RM21976, RM248 |
| 8 | RM310, RM6999, RM404, RM2339, RM331, RM44 | RM152, RM25, RM339, RM331, RM342, RM6999 |
| 9 | RM23788, RM729, RM41, RM1328, RM242, RM3025 | RM23865, RM41, RM13912, RM72, RM1328, RM24386 |
| 10 | RM474, RM3773, RM7217, RM228, RM222 | RM7217, RM3773, RM228, RM216, RM474 |
| 11 | RM332, RM1233, RM144, RM26334, RM287, RM21, RM254, RM1812, RM1233, RM224 | RM287, RM26334, RM1812, RM332, RM21, RM254, RM1233, RM224, RM144 |
| 12 | RM28048, RM19, RM3813, RM1302, RM17 | RM1302, RM28102, RM5196, RM314, RM26 |

**Table S2 |** Details of recurrent parent genome recovery in ADT 43, and ASD 16 cross combinations

| **Genotype** | **Recurrent genome recovery**  **(G) %** |
| --- | --- |
| **ADT 43 × IRBB60** | |
| **BC1F1** | |
| IL-2-1-33 | 77.08 |
| IL-2-2-53 | 76.04 |
| **BC2F1** | |
| IL-2-10-4 | 88.54 |
| IL-2-12-1 | 88.54 |
| IL-2-18-3 | 87.5 |
| **BC_3_F1** | |
| IL-2-6-61 | 94.26 |
| IL-2-8-34 | 94.04 |
| **Improved ADT 43 (*xa5*, *xa13*, and *Xa21*) × Tetep** | |
| **BC1F1** | |
| IPL-2-5-12 | 76.19 |
| IPL-2-8-19 | 77.11 |
| IPL-10-4 | 76.33 |
| **BC2F1** | |
| IPL-2-11-13 | 86.47 |
| IPL-2-2-41 | 88.21 |
| IPL-2-6-2 | 87.62 |
| IPL-2-7-21 | 85.61 |
| **BC_3_F1** | |
| IPL-2-8-22 | 93.88 |
| IPL-2-5-1 | 94.92 |

| **Genotype** | **Recurrent genome recovery**  **(G) %** |
| --- | --- |
| **ADT 16 × IRBB60** | |
| **BC1F1** | |
| IL-1-2-2 | 75.60 |
| IL-1-5-6 | 76.82 |
| IL-1-8-18 | 76.56 |
| **BC2F1** | |
| IL-1-7-2 | 87.80 |
| IL-1-5-4 | 89.02 |
| **BC_3_F1** | |
| IL-1-3-34 | 93.85 |
| IL-1-3-57 | 94.16 |
| IL-1-5-58 | 94.96 |
| **Improved ASD 16 (*xa5*, *xa13*, and *Xa21*) × Tetep** | |
| **BC1F1** | |
| IPL-1-3-2 | 75.59 |
| IPL-1-5-11 | 77.24 |
| IPL-1-6-2 | 75.13 |
| **BC2F1** | |
| IPL-1-6-5 | 88.27 |
| IPL-1-8-9 | 87.81 |
| IPL-1-9-2 | 87.66 |
| IPL-1-3-11 | 86.81 |
| **BC_3_F1** | |
| IPL-1-9-43 | 94.29 |
| IPL-1-8-33 | 93.98 |

| **Genotype** | **HP (%)** | **MP (%)** | **HRR**  **(%)** | **KL (mm)** | **KB (mm)** | **KLBR** | **MRL (mm)** | **MRB (mm)** | **KLAC (mm)** | **KBAC (mm)** | **LER** |
| --- | --- | --- | --- | --- | --- | --- | --- | --- | --- | --- | --- |
| ACM 18012 | 71.32 | 64.97 | 61.22 | 6.4 | 2.0 | 3.2 | 5.4 | 1.9 | 8.9 | 2.3 | 1.64 |
| ACM 18013 | 71.87 | 63.98 | 62.12 | 6.4 | 2.0 | 3.2 | 5.0 | 2.0 | 9.0 | 2.1 | 1.88 |
| ACM 18014 | 72.65 | 65.47 | 60.18 | 6.6 | 1.9 | 3.4 | 5.0 | 1.8 | 9.0 | 2.3 | 1.96 |
| ACM 18015 | 75.89 | 64.58 | 61.35 | 6.4 | 2.0 | 3.2 | 6.0 | 1.9 | 9.1 | 2.3 | 1.81 |
| ACM 18016 | 71.98 | 63.05 | 60.11 | 6.4 | 2.0 | 3.2 | 6.0 | 1.9 | 8.9 | 2.1 | 1.75 |
| ACM 18019 | 71.20 | 63.94 | 60.26 | 6.6 | 1.8 | 3.6 | 5.6 | 1.6 | 9.0 | 2.2 | 1.92 |
| ACM 18020 | 72.64 | 64.00 | 61.27 | 6.6 | 2.0 | 3.3 | 6.0 | 1.9 | 8.9 | 2.1 | 1.73 |
| ACM 18022 | 72.79 | 65.13 | 61.62 | 6.4 | 2.0 | 3.2 | 5.8 | 1.9 | 9.2 | 2.2 | 1.68 |
| ACM 18242 | 79.12 | 72.11 | 63.11 | 5.4 | 2.6 | 2.1 | 5.1 | 2.4 | 8.3 | 3.5 | 1.68 |
| ACM 18243 | 78.22 | 72.20 | 63.27 | 5.5 | 2.7 | 2.1 | 5.1 | 2.5 | 8.4 | 3.4 | 1.66 |
| ACM 18244 | 78.54 | 71.77 | 62.53 | 5.3 | 2.6 | 2.1 | 5.1 | 2.4 | 8.3 | 3.5 | 1.65 |
| ACM 18245 | 78.38 | 72.07 | 63.11 | 5.3 | 2.6 | 2.1 | 5.1 | 2.4 | 8.4 | 3.5 | 1.67 |
| ACM 18248 | 78.47 | 72.44 | 64.14 | 5.4 | 2.6 | 2.1 | 5.0 | 2.4 | 8.3 | 3.5 | 1.66 |
| ACM 18249 | 78.23 | 72.11 | 63.19 | 5.4 | 2.6 | 2.1 | 5.1 | 2.4 | 8.3 | 3.5 | 1.67 |
| ACM 18250 | 78.23 | 72.03 | 63.12 | 5.4 | 2.6 | 2.1 | 5.1 | 2.4 | 8.4 | 3.5 | 1.66 |
| ACM 18245 | 79.12 | 72.09 | 64.25 | 5.4 | 2.6 | 2.1 | 5.0 | 2.4 | 8.4 | 3.5 | 1.65 |
| ACM 18242 | 78.15 | 72.07 | 63.87 | 5.4 | 2.6 | 2.0 | 5.0 | 2.4 | 8.4 | 3.4 | 1.66 |
| ASD 16 | 78.19 | 71.70 | 62.14 | 5.4 | 2.6 | 2.0 | 5.0 | 2.4 | 8.3 | 3.5 | 1.65 |
| ADT 43 | 72.5 | 65.22 | 59.87 | 6.3 | 2.0 | 3.1 | 6.0 | 1.8 | 8.8 | 2.2 | 1.54 |
| **SE** | 1.04 | 0.37 | 0.24 | 0.35 | 0.03 | 0.03 | 0.07 | 0.04 | 0.02 | 0.02 | 0.03 |
| **CD (5%)** | 3.07 | 1.1 | 0.7 | 1.03 | 0.1 | 0.09 | 0.21 | 0.12 | 0.06 | 0.06 | 0.11 |

**Table S3 |** Evaluation of quality traits of improved pyramided lines at BC_3_F_3_ generation
